# Socioeconomic Position and Type 2 Diabetes: The Mediating Role of Psychosocial Work Environment- the Maastricht Study

**DOI:** 10.3389/ijph.2023.1606036

**Published:** 2023-09-07

**Authors:** Bengisu Sezer, Annemarie Koster, Jeroen Albers, Rachelle Meisters, Miranda Schram, Simone Eussen, Nicole Dukers, Angelique de Rijk, Coen Stehouwer, Hans Bosma

**Affiliations:** ^1^ Department of Social Medicine, Faculty of Health, Medicine and Life Sciences, Maastricht University, Maastricht, Netherlands; ^2^ Institute of Care and Public Health Research, Faculty of Health, Medicine and Life Sciences, Maastricht University, Maastricht, Netherlands; ^3^ Department of Internal Medicine, Faculty of Health, Medicine and Life Sciences, Maastricht University, Maastricht, Netherlands; ^4^ School for Cardiovascular Diseases, Faculty of Health, Medicine and Life Sciences, Maastricht University, Maastricht, Netherlands; ^5^ School for Mental Health and Neuroscience, Faculty of Health, Medicine and Life Sciences, Maastricht University, Maastricht, Netherlands; ^6^ Heart and Vascular Center, Maastricht University Medical Centre, Maastricht, Netherlands; ^7^ Department of Epidemiology, Faculty of Health, Medicine and Life Sciences, Maastricht University, Maastricht, Netherlands; ^8^ Department of Health Promotion, Faculty of Health, Medicine and Life Sciences, Maastricht University, Maastricht, Netherlands; ^9^ South Limburg Medical Health Service (GGD South Limburg), Heerlen, Netherlands

**Keywords:** socioeconomic inequalities, psychosocial work environment, type 2 diabetes mellitus, mediation, job demand/control/support

## Abstract

**Objective:** We examined the association between low socioeconomic position (SEP) and Type 2 Diabetes Mellitus (T2DM), and the mediating role of psychosocial work environment by using counterfactual mediation analysis.

**Methods:** Data from 8,090 participants of The Maastricht Study were analysed. SEP indicators (education, income, occupation), self-reported psychosocial work stressors, (pre)diabetes by oral glucose tolerance test were measured at baseline. Incident T2DM was self-reported per annum up to 9 years. Cox regression and causal mediation analyses were performed.

**Results:** 2.8% (*N* = 172) of the participants without T2DM at baseline reported incident T2DM. People with lower SEP more often had prevalent T2DM (e.g., education OR = 2.49, 95% CI: 2.16–2.87) and incident T2DM (e.g., education HR = 2.21, 95% CI: 1.53–3.20) than higher SEP. Low job control was associated with prevalent T2DM (OR = 1.44 95% CI: 1.25–1.67). Job control partially explained the association between income and prevalent T2DM (7.23%). Job demand suppressed the associations of education and occupation with prevalent T2DM. The mediation models with incident T2DM and social support were not significant.

**Conclusion:** Socioeconomic inequalities in T2DM were present, but only a small part of it was explained by the psychosocial work environment.

## Introduction

Socioeconomic position (SEP) has significant influences on health outcomes [1]. Previous studies have shown that people with lower income or lower education levels are more likely to develop type 2 diabetes mellitus (T2DM) than people with higher income and higher education levels [2–4]. While researchers acknowledge low SEP as a risk factor for poor health outcomes, the ways low SEP influences T2DM need further explanations. Upstream factors in T2DM such as environmental stressors are understudied compared to downstream factors such as lifestyle behaviours. One of these upstream factors can be psychosocial work stress which might account for some of the variance in this relationship [5, 6].

A stressful work environment poses a serious threat for individual health. Previous studies demonstrated a link between psychosocial work environment and various health outcomes such as health functioning [7] and cardiovascular diseases [8]; however, there is no accordance in the literature about the role of psychosocial work stress in T2DM. While some studies supported that work-related stress is a risk factor for developing diabetes [6, 9], other researchers failed to detect a statistically significant relationship [10–12]. These studies vary in their operationalization of psychosocial stress, target population, and statistical methods, which might partially explain the inconsistent findings. Furthermore, the literature remains inconclusive regarding the potential sex-dependent effects of psychosocial work environment on T2DM [6, 10]. More comprehensive research on the role of job stress is needed to explore the work-related factors in relation to T2DM status.

People with higher levels of SEP are expected to have larger autonomy, higher psychological demands, and more supportive social relationships at work [13]. On the other hand, the asymmetry between demand and autonomy, job strain, is considered to be more common in the lower SEP groups [14]. We adopted Karasek’s Job Demand-Control-Support Model as the theoretical framework [15, 16] to investigate the role of the psychosocial work environment and SEP in T2DM.

Although socioeconomic factors and work environment were studied in relation to health outcomes, less is known about whether work environment can explain the adverse health consequences of lower SEP levels. Previously, researchers suggested exploring the mediating roles of environmental factors, which can be the work environment, in SEP and health relationship [2, 17]. Therefore, we here evaluated psychosocial work environment as a pathway between SEP and T2DM and aimed to investigate its role as a mediator. Exploring the environmental factors in the T2DM exposome will contribute to more elaborate interventions as well as a more comprehensive understanding of the disease.

In this project, we use data from The Maastricht Study to investigate the association between SEP with prevalent prediabetes, prevalent and incident T2DM, and the potential mediating role of psychosocial work environment. First, we hypothesize that people with lower levels of SEP are more likely to have (pre)diabetes and develop T2DM. Second, we hypothesize that people in a stressful work environment that is high levels of job demands, low levels of job control, and low levels of social support are more likely to have prediabetes, prevalent and incident T2DM. Lastly, we expect to support the mediating role of psychosocial work environment in the relationship of SEP and T2DM. Supplementary Figure S1 illustrates the theoretical model for our hypotheses (see Supplementary Material).

## Methods

### Study Population

We used data from The Maastricht Study, an observational prospective population-based cohort study. The rationale and methodology have been described previously [18]. In brief, the study focuses on the etiology, pathophysiology, complications, and comorbidities of T2DM and is characterized by an extensive phenotyping approach. Eligible participants were aged between 40 and 75 years and living in the southern part of the Netherlands. Participants were recruited through mass media campaigns and from the municipal registries and the regional Diabetes Patient Registry. Recruitment was stratified according to known T2DM status with an oversampling of individuals with T2DM for reasons of efficiency. The current study includes cross-sectional data from 9,188 participants who completed the baseline survey between November 2010 and October 2020. Supplementary Figure S2 illustrate the number of participants per year (see Supplementary Material). The examinations of each participant were performed within a time window of 3 months. The participants were followed for a maximum of 9 years after the baseline. Annual follow-up data were available for 89.9% (year 1), 79.1% (year 2), 70.2 (year 3), 63.5% (year 4), 62.7% (year 5), 51.5% (year 6), 42.3% (year 7), 26.6% (year 8), and 8.8% (year 9) of the participants. The response rate decreased over the years of follow-up date due to the ongoing follow-up measurements during the time of our study. Supplementary Figure S3 illustrates the median follow-up years per baseline participation year (see Supplementary Material).

The final sample consisted of 8,090 participants [*M*_age_ = 59.3 (±8.7); 50.1% women] after the exclusion of people with diabetes other than T2DM, and people with missing values in education and psychosocial work environment for the initial sample. Supplementary Figure S4 illustrates a flow diagram representing the participant inclusion process (see Supplementary Material). For the longitudinal analyses, the study sample consisted of 6,134 [*M*_age_ = 58.6 (±8.6); 54.9% women] participants after the additional exclusion of people with prevalent T2DM, and people without any available follow-up data. Additionally, people with missing values in SEP indicators were excluded for a complete case analysis, creating separate sample sizes for each SEP model of education, income (cross-sectional *N* = 6,637, longitudinal *N* = 5,070), and occupation (cross-sectional *N* = 3,507, longitudinal *N* = 2,650).

### Measurements

#### Diabetes Status

Prediabetes and prevalent T2DM was defined in accordance with WHO 2006 criteria [19]. All participants underwent a standardized seven-point oral glucose tolerance test (OGTT) after overnight fasting. Blood samples were collected at baseline and 15, 30, 45, 60, 90, and 120 min after the consumption of a 75 g glucose drink. Participants who were insulin-dependent or with a fasting glucose level higher than 11.0 mmol/L (as determined by finger prick) did not undergo this test. Participants on diabetes medication and without type 1 diabetes were also considered to have T2DM.

#### Diabetes Survival Time

T2DM status after baseline was assessed by self-reported follow-up questionnaires annually. Participants were asked “Has a doctor told you that you have diabetes in the last 12 months? [yes/no/I don’t know].” “I don’t know” was recoded as a missing value. Survival time for diabetes cases was computed as the midpoint between the follow-up date at which the event was reported and the previous follow-up date. Survival time for censored cases was computed as the time of the last available follow-up date. For 19 participants with incident T2DM who had missing values in the previous follow-up point, we subtracted 182.6 days (half a year) from the report date. Lastly, we computed the days between baseline measurement and survival time, and divided by 365.25 to compute survival time in years.

#### Socioeconomic Position

##### Education

Participants were asked to indicate the highest education level they had completed in categories (1 = None, 2 = Primary educational level, 3 = Lower vocational education, 4 = Intermediate general secondary education, 5 = Intermediate vocational education, 6 = Higher general secondary education, 7 = Higher vocational education, 8 = University education, 9 = Uncompleted primary educational level, 10 = Other, 11 = I don’t know). Categories 10 (Other) and 11 (do not know) were coded as missing values. For continuous analyses, “uncompleted primary education [9]” was recoded as the second answer category [2]. For categorical analyses, we categorized education level as low [1–5], intermediate [6, 7], and high [8, 9] approximately creating tertiles.

##### Income

Participants were asked to indicate their net monthly household income in 19 categories ranging from less than 750 euros to more than 5,000 euros. Then, the reported household income was divided by the square root of household size to achieve an equivalent value per person [20, 21]. We categorized the final income as low, intermediate, and high based on tertiles.

##### Occupation

Participants were asked to describe their current occupation in an open-ended question. The open-ended question was coded to International Standard Classification of Occupations 2008 (ISCO-08) categories by a trained coder [22]. Subsequently, the ISCO codes were converted to the International Socio-Economic Index of Occupational Status (ISEI-08) to indicate occupational class status forming a continuous scale. We categorized the ISEI-08 classifications as low, intermediate, and high occupation status based on tertiles.

#### Psychosocial Work Environment

The psychosocial work environment was defined by three components of the Job Demand-Control-Support Model [15, 16] and measured by 10 items on a Likert scale, 1-Always to 4-Never. Participants who were unemployed at the time of the baseline measurements answered the questionnaire based on their previous jobs. The scale consisted of 3-item job demand (e.g., “Did/do you have too little time to perform your job sometimes?”), 5-item job control (e.g., “Could/can you decide when you start/stop and take breaks at your work?”), and 3-item social support at job (e.g., “Did/does your manager/management support you enough at work?”) measures. We calculated composite scores for each factor and categorized as low, intermediate, and high based on tertiles.

### Statistical Analysis

Baseline characteristics of the study sample were compared across groups of glucose metabolism status (normal glucose tolerance, prediabetes, T2DM). To investigate the relationships of SEP and psychosocial work environment indicators with T2DM, we conducted separate multinomial logistic regression analyses with cross-sectional data and Cox proportional hazards regression analyses with longitudinal data controlling for age and sex. The proportional hazards assumption was tested by statistical tests and visualisation before the survival analysis and was not violated. Chi-square tests were used to test the relationship between SEP and psychosocial work environment. Subsequently, we conducted causal mediation analyses by using a counterfactual framework [23]. By using a simulation-based approach [24, 25], we computed the mediation models with 1,000 iterations, basic percentile intervals, and loglogistic distribution for the survival models. We accounted for the possibility of an interaction between exposures and mediators. We used the continuous versions of SEP and psychosocial work environment factors contrasting the mean score of low and high SEP groups.

For further sensitivity analysis, we analysed if the associations of SEP and psychosocial work environment factors with T2DM are dependent on age, sex, or currently (un)employed by separately including interaction terms to our regression models to test the robustness of our findings. We explored job strain (the imbalance of job control and job demand) and iso-strain (job strain including social support) by investigating the 2-way and 3-way interactions. All analyses were conducted in the statistical software, RStudio [26] by using *tidyverse* [27] for data cleaning and manipulation, *nnet* [28] for logistic regression analysis, *mediation* [24] for mediation analysis, and *survival* [29] for survival analysis.

## Results

Of the participants, 20.1% had T2DM at baseline [Mage = 61.6 (±7.8); 31.7% women] (not tabulated). During a median follow-up period of 6.2 years, 172 (2.8%) participants reported T2DM [Mage = 61.1 (±8.1); 43% women; 71.5% prediabetes at baseline]. Of people with prediabetes at baseline, 123 (10.7%) reported T2DM during follow-up. Table 1 shows the demographics of participants and the distribution of SEP indicators and psychosocial work stressors by T2DM status. People with lower levels of SEP more often had prevalent T2DM than people with a higher SEP. For example, 28.1% of people with low education were diagnosed with prevalent T2DM in comparison to 14.3% of people with high education. Similarly, higher incidence rates of T2DM were observed in people with lower levels of SEP than people with higher levels of SEP. For example, 3.8% of people with a low income were diagnosed with T2DM during the follow-up period in comparison with 1.6% of people with a high income.

**TABLE 1:** Demographics, socioeconomic position indicators, and psychosocial work environment factors by type 2 diabetes mellitus status (The Maastricht Study, Limburg, The Netherlands, 2010–2020).

	Cross-sectional				Longitudinal		
	Normal Glucose Metabolism	Prediabetes	Prevalent T2DM	*p*-value	No Incident T2DM	Incident T2DM	*p*-value
Age	57.6 (8.61)	61.8 (8.2)	61.6 (7.8)	<0.001	58.5 (8.63)	61.1 (8.12)	<0.001
Sex				<0.001			0.002
Female	2,979 (73.6%)	550 (13.6%)	517 (12.8%)		3,294 (97.8%)	74 (2.2%)	
Male	2,270 (56.1%)	662 (16.4%)	1,112 (27.5%)		2,668 (96.5%)	98 (3.5%)	
Education				<0.001			<0.001
High	2,394 (72%)	454 (13.7%)	476 (14.3%)		2,657 (98.1%)	51 (1.9%)	
Intermediate	1,489 (65.9%)	322 (14.3%)	448 (19.8%)		1,669 (97.2%)	48 (2.8%)	
Low	1,366 (54.5%)	436 (17.4%)	705 (28.1%)		1,636 (95.7%)	73 (4.3%)	
Income				<0.001			<0.001
High	1,540 (70.1%)	318 (14.5%)	338 (15.4%)		1,628 (98.4%)	26 (1.6%)	
Intermediate	1,301 (69.3%)	257 (13.7%)	320 (17%)		1,551 (97.4%)	41 (2.6%)	
Low	1,515 (59.1%)	404 (15.8%)	644 (25.1%)		1,754 (96.2%)	70 (3.8%)	
Occupation				<0.001			0.082
High	848 (71%)	175 (15.3%)	171 (14.3%)		955 (98.2%)	18 (1.8%)	
Intermediate	763 (66.1%)	177 (15.3%)	215 (18.6%)		856 (97.6%)	21 (2.4%)	
Low	675 (58.3%)	190 (16.4%)	293 (25.3%)		770 (96.2%)	30 (3.8%)	
Job Control				0.171			0.662
High	2,028 (64.6%)	475 (15.1%)	638 (20.3%)		2,308 (97.3%)	63 (2.7%)	
Intermediate	1,718 (66.5%)	382 (14.8%)	484 (18.7%)		1,936 (97.3%)	54 (2.7%)	
Low	1,503 (63.6%)	355 (15%)	507 (21.4%)		1,718 (96.9%)	172 (2.8%)	
Job Demand				0.016			0.417
High	1,866 (64.7%)	393 (13.6%)	627 (21.7%)		2,074 (97.1%)	61 (2.9%)	
Intermediate	2,236 (65.4%)	538 (15.7%)	645 (18.9%)		2,580 (97.5%)	67 (2.5%)	
Low	1,147 (64.3%)	281 (15.7%)	357 (20%)		1,308 (96.7%)	44 (3.3%)	
Job Support				0.001			0.969
High	1,705 (63%)	424 (15.7%)	577 (21.3%)		1,959 (97.2%)	56 (2.8%)	
Intermediate	2,155 (66.9%)	486 (15.1%)	581 (18%)		2,459 (97.2%)	70 (2.8%)	
Low	1,389 (64.2%)	302 (14%)	471 (21.8%)		1,544 (97.1%)	46 (2.9%)	

Note. Means (and standard deviations) and *p*-values for Anova tests are reported for continuous variables and number of people (and row percentages) and *p*-values for chi-squared tests are reported for categorical variables.

Table 2 shows that SEP indicators were associated with prediabetes, prevalent and incident T2DM when controlled for age and sex. Compared to people with a higher level of education, people with a lower level of education had an increased odds of having prediabetes (OR = 1.49, 95% CI 1.28, 1.74) and prevalent T2DM (OR = 2.49, 95% CI 2.16, 2.87). Similarly, people with a lower level of income and a lower level of occupation had an increased odds of having prediabetes (income: OR = 1.35, 95% CI 1.14, 1.59; occupation: OR = 1.50 95% CI 1.19, 1.90) and prevalent T2DM (income: OR = 2.15, 95% CI 1.84, 2.52; occupation: OR = 2.63, 95% CI 2.10, 3.30) compared to people with a higher level of income and occupation. The longitudinal analysis also showed that lower levels of SEP significantly increased the risk of incident T2DM. Compared to people with higher levels of education, people with a lower level of education had an increased risk of incident T2DM (HR = 2.21, 95% CI 1.53, 3.20). Similarly, people with a lower level of income or a lower level of occupation had an increased risk of incident T2DM (income: HR = 2.53, 95% CI 1.61, 3.98; occupation: HR = 2.26, 95% CI 1.26, 4.08), compared to those with a higher level of income or occupation. A lower level of job control was statistically significantly associated with prevalent T2DM (OR = 1.44, 95% CI 1.25, 1.67), but not with incident T2DM (HR = 1.33, 95% CI 0.92, 1.94) compared to a higher level of job control. Job demand and social support were not statistically significantly associated with prediabetes, prevalent and incident T2DM.

**TABLE 2:** Associations of socioeconomic position indicators and psychosocial work environment with prediabetes, and prevalent and incident type 2 diabetes (The Maastricht Study, Limburg, The Netherlands, 2010–2020).

	Cross-sectional			Longitudinal	
		Prediabetes	T2DM		Incident T2DM
	*N*	OR (95% CI)	OR (95% CI)	*N*	HR (95% CI)
Education					
High	3,324			2,708	
Intermediate	2,259	1.27** (1.08, 1.49)	1.75*** (1.51, 2.04)	1,717	1.58* (1.07, 2.35)
Low	2,507	1.49*** (1.28, 1.74)	2.49*** (2.16, 2.87)	1,709	2.21*** (1.53, 3.20)
Income					
High	2,196			1,824	
Intermediate	1,878	1.05 (0.87, 1.26)	1.28** (1.08, 1.53)	1,592	1.58* (1.07, 2.35)
Low	2,563	1.35*** (1.14, 1.59)	2.15*** (1.84, 2.52)	1,654	2.53*** (1.61, 3.98)
Occupation					
High	1,194			973	
Intermediate	1,155	1.23 (0.97, 1.55)	1.59*** (1.26, 2.01)	877	1.42 (0.75, 2.66)
Low	1,158	1.50*** (1.19, 1.90)	2.63*** (2.10, 3.30)	800	2.26** (1.26, 4.08)
Job Control					
High	3,141			2,371	
Intermediate	2,584	1.02 (0.88, 1.19)	1.06 (0.92, 1.22)	1,990	1.09 (0.75, 1.57)
Low	2,365	1.12 (0.96, 1.32)	1.44*** (1.25, 1.67)	1,773	1.33 (0.92, 1.94)
Job Demand					
Low	1,785			1,352	
Intermediate	3,419	1.04 (0.88, 1.23)	0.90 (0.77, 1.06)	2,647	0.77 (0.53, 1.14)
High	2,886	0.95 (0.80, 1.14)	1.09 (0.93, 1.28)	2,135	0.89 (0.60, 1.33)
Job Support					
High	2,706			2015	
Intermediate	3,222	0.95 (0.82, 1.10)	0.85* (0.74, 0.97)	2,529	1.02 (0.72, 1.45)
Low	2,162	0.92 (0.78, 1.09)	1.10 (0.95, 1.27)	1,590	1.09 (0.74, 1.61)

Note. *p < 0.05 **p < 0.01 ***p < 0.001.

Note. All models were adjusted for age and sex.

Note. High SEP category was taken as the reference group.

Note. Reference group for the cross-sectional analysis is the normal glucose level category.

Table 3 shows that SEP indicators were statistically significantly associated with psychosocial work environment. For instance, 43.5% of the people with a low level of education had a low level of job control compared to 17.8% of the people with a high level of education. 33.5% of people with a low income had a high level of job demand compared to 40.6% of people with a high income. People with lower SEP levels had statistically significantly more often lower levels of job control and social support, and less often had higher levels of job demand, consistently across all SEP indicators. The results of the regression analysis that are presented in Supplementary Table S1 were also in line with these findings (see Supplementary Material).

**TABLE 3:** The sample sizes and percentages of socioeconomic position indicators by the risk group of the psychosocial work environment factors (The Maastricht Study, Limburg, The Netherlands, 2010–2020).

	Low job control		High job demand		Low job support	
	*N* (%)	*p*-value	*N* (%)	*p*-value	*N* (%)	*p*-value
Education		<0.001		<0.001		<0.001
High	593 (17.8%)		1,358 (40.9%)		812 (24.4%)	
Intermediate	682 (30.2%)		803 (35.5%)		594 (26.3%)	
Low	1,090 (43.5%)		725 (28.9%)		756 (30.2%)	
Income		<0.001		<0.001		<0.001
High	351 (16%)		892 (40.6%)		457 (20.8%)	
Intermediate	464 (24.7%)		678 (36.1%)		429 (22.8%)	
Low	1,040 (40.6%)		859 (33.5%)		824 (32.1%)	
Occupation		<0.001		<0.001		<0.001
High	239 (20%)		513 (43%)		293 (24.5%)	
Intermediate	254 (22%)		416 (36%)		266 (23%)	
Low	492 (42.5%)		338 (29.2%)		365 (31.5%)	

Note. Row percentages were presented.

Note. Only the risk categories of psychosocial work environment were included in the table.

Table 4 presents the results of the mediation analyses by showing the indirect effects of and proportion mediated by the mediator. We found that 7.23% of the relationship between income and prevalent T2DM was explained by job control. Additionally, the results indicated a suppression effect in two different models with a negative indirect effect and a positive direct effect. We found that the relationship between education and prevalent T2DM and the relationship between occupation and prevalent T2DM were suppressed by job demands. In other words, low job demands decreased the negative influence of low education and low occupation on T2DM. Social support at work did not mediate any of the models. Furthermore, we did not find the mediating effects of job control or job demand in the models of prediabetes and incident T2DM as the outcome. We also accounted for the potential interaction between SEP and psychosocial work environment indicators; however, the interaction terms were not significant. Thus, the mediation models were conducted excluding an exposure and mediator interaction.

**TABLE 4:** Indirect effects and proportions mediated of causal mediation models (The Maastricht Study, Limburg, The Netherlands, 2010–2020).

	Education			Income			Occupation		
	Prediabetes	Prevalent T2DM	Incident T2DM	Prediabetes	Prevalent T2DM	Incident T2DM	Prediabetes	Prevalent T2DM	Incident T2DM
Job Control									
Indirect Effect	−0.001 (−0.01, 0.01)	0.01 (−0.002, 0.01)	8.81 (−79.67, 104.09)	−0.001 (−0.01, 0.01)	0.01* (0.001, 0.01)	32.30 (−130.66, 216.86)	−0.002 (−0.01, 0.01)	0.005 (−0.005, 0.01)	−28.58 (−222.22, 106.62)
Proportion Mediated	−0.02 (−0.15, 0.10)	0.03 (−0.01, 0.08)	−0.02 (−0.17, 0.13)	−0.01 (−0.22, 0.16)	0.07* (0.01, 0.14)	−0.04 (−0.23, 0.13)	−0.03 (−0.32, 0.17)	0.03 (−0.03, 0.09)	0.05 (−0.19, 0.23)
Job Demand									
Indirect Effect	0.001 (−0.003, 0.01)	−0.01** (−0.01, 0)	10.61 (−41.43, 68.39)	0.002 (−0.001, 0)	−0.002 (−0.004, 0)	−4.78 (−77.63, 43.24)	0.001 (−0.01, 0.01)	−0.01* (−0.01, 0)	−15.93 (−187.08, 108.07)
Proportion Mediation	0.02 (−0.05, 0.10)	−0.04** (−0.06, −0.01)	−0.02 (−0.1, 0.07)	0.04 (−0.02, 0.14)	−0.02 (−0.04, 0)	0.006 (−0.05, 0.06)	0.02 (−0.12, 0.18)	−0.04* (−0.08, 0)	0.03 (−0.14, 0.21)
Job Support									
Indirect Effect	−0.0004 (−0.001, 0)	0.0004 (−0.0004, 0)	0.9 (−8.92, 14.04)	−0.001 (−0.004, 0)	0.0003 (−0.002, 0)	−2.70 (−63.53, 47.76)	−0.0002 (−0.002, 0)	0.001 (−0.001, 0)	1.79 (−12.99, 17.64)
Proportion Mediated	−0.01 (−0.02, 0.01)	0.002 (−0.002, 0.01)	−0.002 (−0.02, 0.01)	−0.03 (−0.11, 0.03)	0.003 (−0.02, 0.03)	0.003 (−0.05, 0.06)	−0.003 (−0.04, 0.02)	0.01 (−0.01, 0.02)	−0.003 (−0.03, 0.02)

Note. *p < 0.05 **p < 0.01 ***p < 0.001.

In sensitivity analyses, we tested if the associations of SEP and psychosocial work environment with T2DM was dependent on age, sex, or current employment status by including 2-way interactions in models. We tested the models by using both continuous and categorical versions of SEP and psychosocial work environment indicators. We observed some interactions effects with age such as the interaction of age with low job control in relation to prevalent T2DM or with intermediate occupation in relation to prediabetes. Five out of 36 interaction terms were significant, but they were not consistent across T2DM status (see Supplementary Table S2). The interaction of sex with SEP and psychosocial work environment was not statistically significant (see Supplementary Table S3). The current employment status also did not interact with continuous or categorical versions of SEP or psychosocial work environment factors. We tested 2-way and 3-way interactions between psychosocial work environment in relation to T2DM status by using both continuous and categorical versions of these variables to investigate job strain and iso-strain. Only two out of 24 interaction terms were significant (see Supplementary Table S4). Due to few numbers of significant interaction terms and inconsistent patterns with T2DM status, we decided not to include job strain or iso-strain in our models. Moreover, we conducted the survival analyses with three different outcomes. First, we used the time of diabetes diagnosis reported by participants, instead of estimating missing values using midpoint imputation. Then, we used a conservative approach that censored participants at the first instance of a missing value, even if they did not report diabetes after the missing value. The results were in line with the main analyses implying robustness of our findings. Although, there were some statistically significant interaction terms, the results did not indicate moderation.

## Discussion

This study is one of the first to test potential mediation of the psychosocial work environment in the relationship of SEP and T2DM by using a counterfactual framework for causal mediation analysis. We first showed that lower levels of SEP were associated with a higher prevalence and incidence of T2DM, and prevalence of prediabetes. Second, our study provided partial evidence for the negative effects of a stressful psychosocial work environment on T2DM by showing that a lower level of job control is associated with prevalent T2DM, but not with incident T2DM. Job demands were not statistically significantly associated with diabetes outcomes. Despite the non-significant association between job demands and T2DM, we could still compute the mediation models [30, 31]. Lastly, the mediation analyses demonstrated that job control partially explained the association between income and prevalent T2DM. Job demand suppressed the relationship of education and occupation with prevalent T2DM.

We found evidence for substantial socioeconomic health inequalities by showing the association between SEP indicators and prediabetes, prevalent and incident T2DM. In line with previous research [1, 32, 33], lower levels of SEP were strongly associated with a higher risk of T2DM. Moreover, people with lower levels of SEP had lower levels of control, demand, and support at the workplace as expected [13]. Our results were similar to previous studies that demonstrated a lack of association between psychosocial work stressors and T2DM [5, 10, 12] while contrasting to those supporting the influence of job stress [9]. We did not find a link between psychosocial work environment and T2DM status in our study, except for the association between low job control and prevalent T2DM. It is possible that job control is one of many stressors in the T2DM exposome, but not important enough to explain socioeconomic differences in diabetes by itself. Job control is more often associated with heart-related diseases [8], thus we can shift to focus to a chronic stress perspective as an effect modifier in the future. As an alternative explanation, job control might interfere with lifestyle factors which can be of interest for future studies. While exploring the pathways between SEP and T2DM, we found that the higher risk of prevalent T2DM among people with lower levels of income was to a small extent due to their lower levels of job control supporting the idea that lower SEP people face unhealthier environmental exposures compared to higher SEP people [34]. However, the environment for low SEP groups was not unhealthy in terms of job demands. Lower levels of job demand appeared to *protect* people with lower levels of education and occupation against T2DM. Because the evidence for the pathways was cross-sectional and psychosocial work environment stressors were not directly associated with T2DM, psychosocial work stressors might not be a major environmental factor in the T2DM exposome.

A strength of the current study is the multifaceted, evidence-based measurement of the psychosocial work environment. The Job Demand-Control-Support model allows researchers to capture various psychosocial stressors at the workplace and a validated model for occupation health research [35]. Our results further advanced this theoretical framework by showing the importance of job control in T2DM and by demonstrating the social gradient in the effects of job demands. Although high job demands pose a risk for health outcomes [35], they were less prevalent in the risk group, low SEP, in our study, creating a protective effect. Additionally, the separate main effects of job demands and job control showed higher importance than job strain in our results. To further advance the literature, future research can incorporate broader aspects of the work environment such as the Effort-Reward Imbalance Model, job insecurity, and overcommitment [36].

Previous researchers discussed the association between psychosocial work environment and T2DM in terms of sex differences in studies both demonstrating [10, 37, 38] and not demonstrating [6, 39] a sex-specific effect. Our sensitivity analyses did not show any sex differences in the relationship of diabetes status and SEP or psychosocial work environment. In a previous study, both women and men responded to work stress similarly, but the difference occurred after work hours [40]. It is possible that non-work-related stressors did not differ among sexes in our study sample. Future studies can incorporate a chronic stress perspective and other types of stressors to understand the (lack of) sex differences in this association.

The current study has valuable contributions to scientific literature and has several strengths. First, The Maastricht Study is a large cohort study allowing us to use a large sample size and to examine the longitudinal association between SEP and T2DM. We presented robust evidence for the link between socioeconomic disparities in T2DM. Second, we built upon previous studies on mediating effects of psychosocial work environment by using advanced statistical techniques. We adopted Vanderweele’s causal mediation framework and ran simulations to test our mediation models [23]. Finally, we conducted sensitivity analyses and multiple interactions to test the robustness of our findings.

It is also important to point out some limitations of our study. First, currently unemployed participants responded to the work environment questionnaires based on their previous jobs. To test for potential effect modification, we included the current employment status (employed vs. unemployed) as an interaction term with psychosocial work environment variables and did not find any significant patterns. Other life-long designs might capture the changes in employment better from a longitudinal perspective in the future. Second, participants reported if they were diagnosed with T2DM in the past 12 months through annual questionnaires, which gave us a large time window for the actual development and report of the event. We tackled this limitation by using the midpoint imputation for the survival outcome. Third, the accuracy of income measurement through self-reporting could be affected by social desirability. To tackle this limitation, participants filled out the question by themselves avoiding the pressure by an interviewer with the possibility of not answering the question if they feel uncomfortable. Further analyses showed midpoint imputation acted similar to the date of the report. Lastly, the small sample size of the occupation models might have posed a risk regarding the interpretation of the work environment results. However, occupation models acted similar to other SEP models.

Our study not only advances scientific knowledge, but also makes a social impact. Demonstrating the influences of upstream environmental factors on T2DM contributes to the destigmatization of this disease. Downstream factors such as diet or exercise were not included in the current study to emphasize the importance of environmental effects in T2DM. Future studies can combine upstream and downstream factors and even study how they influence each other. For example, a stressful work environment might be causing unhealthy coping mechanisms like smoking [41]. The external factors in the T2DM exposome should be taken into account while creating interventions [42]. Decision-makers such as municipalities or employers should consider the environmental influences while creating public health policies. Although our results do not suggest practical implications in terms of work stressors to decrease T2DM diagnosis, we provide some evidence for the role of work stressors. The literature could benefit from more qualitative and quantitative research on other types of work stressors to develop workplace interventions for improving health.

To conclude, our findings provide modest evidence for work environmental injustice in the exposome of T2DM. The substantial higher risk of diabetes in low SEP groups is to a small extent explained by these groups having lower control at work. Furthermore, lower psychosocial job demands showed a protective effect on people with low SEP against T2DM. Hence, our findings suggest that work stressors might not be a major target for tackling socioeconomic inequalities in T2DM. There is a need for further studies exploring whether or not there is environmental injustice in the wider upstream exposome of T2DM.
