# Supplementary material for: Socioeconomic Position and Type 2 Diabetes: The Mediating Role of Psychosocial Work Environment- the Maastricht Study
Source: Int J Public Health. 2023 Sep 7;68:1606036. doi: 10.3389/ijph.2023.1606036 (PMC10511755; doi:10.3389/ijph.2023.1606036)
Supplement: Supplementary file 1 [file DataSheet1.docx]

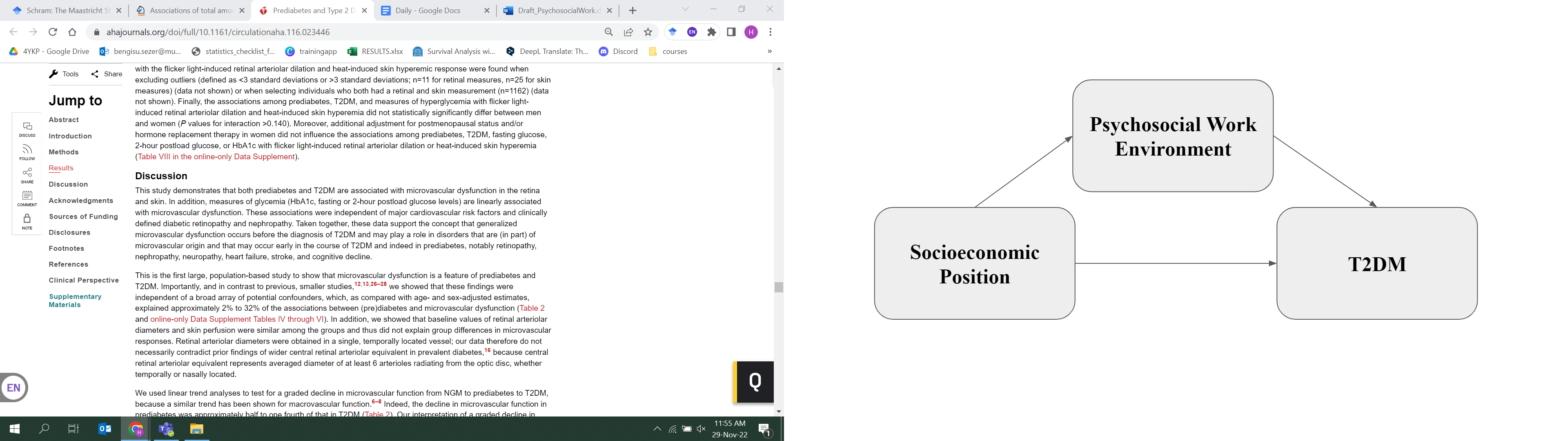


Figure S1. The theoretical model for the mediating role of psychosocial work environment in the relationship of socioeconomic position and type 2 diabetes mellitus.


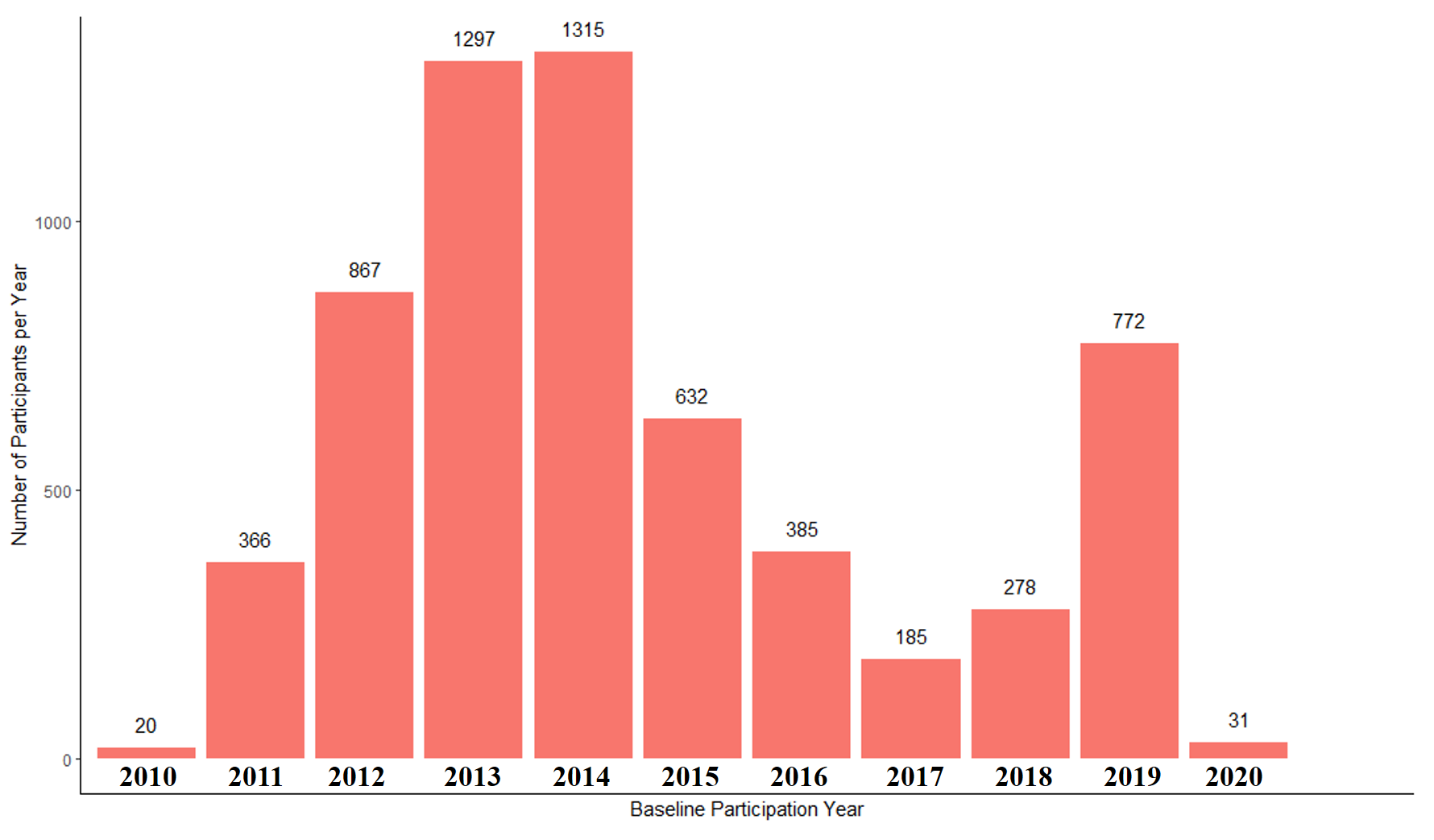


Figure S2. Number of participants per baseline participation year,


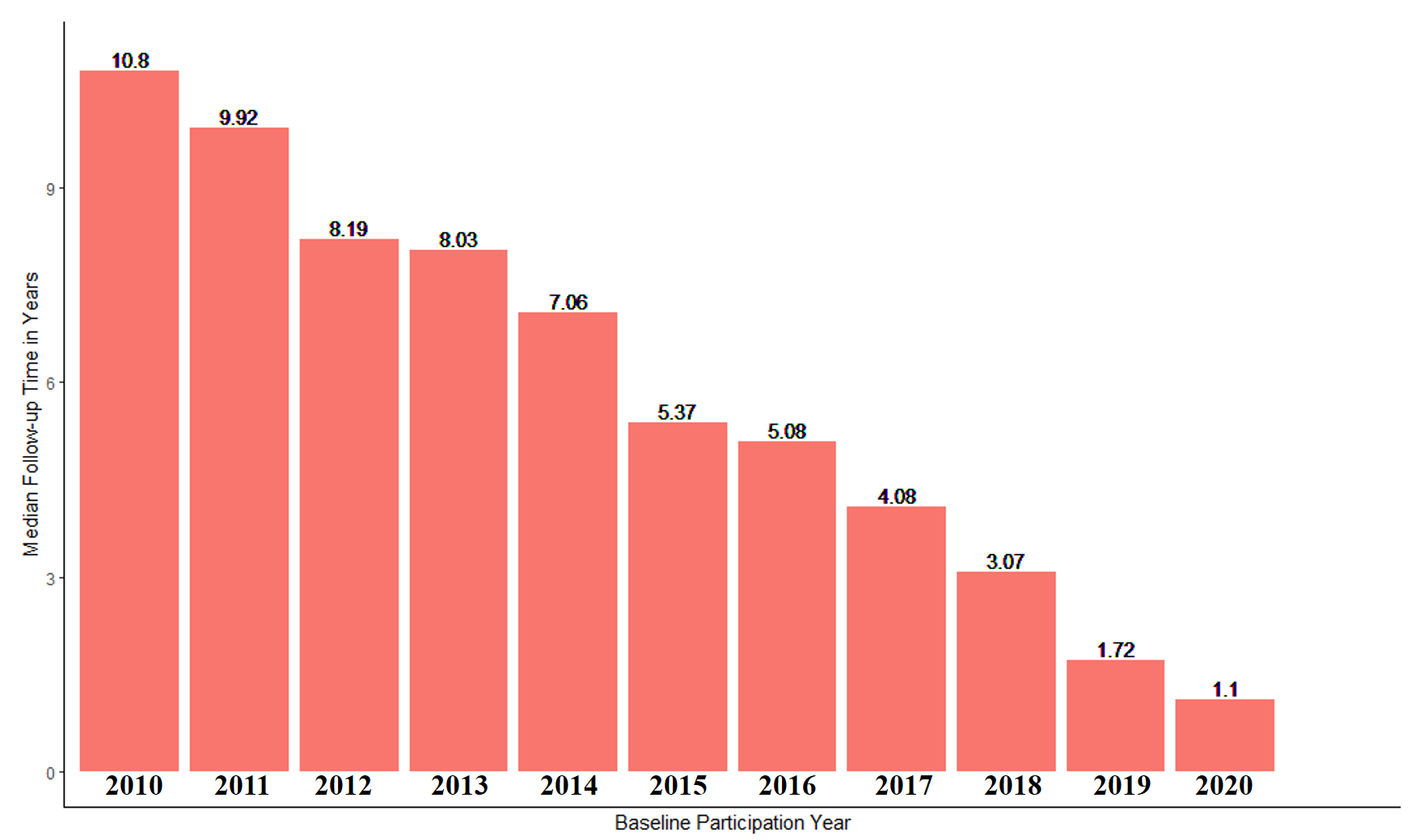


Figure S3. Median follow-up time in years per baseline participation year.


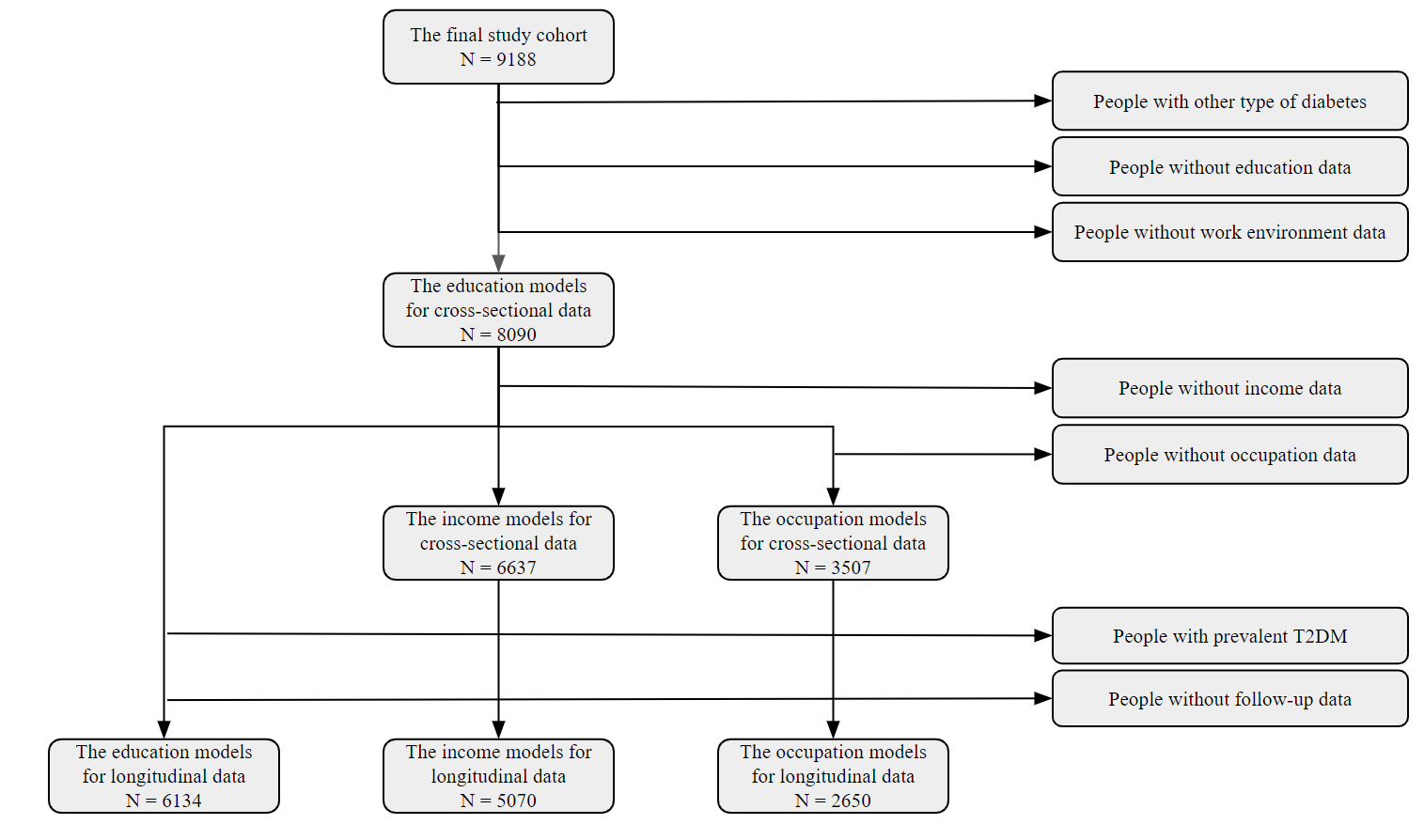


Figure S4. Exclusion criteria.

Table S1. The association of socioeconomic position and psychosocial work environment.

|  | Job Control | | | Job Demand | | | Job Support | | |
| --- | --- | --- | --- | --- | --- | --- | --- | --- | --- |
|  | High | Intermediate | Low | Low | Intermediate | High | High | Intermediate | Low |
|  | *REF* |  |  | *REF* |  |  | *REF* |  |  |
| Education |  |  |  |  |  |  |  |  |  |
| High | *REF* |  |  |  |  |  |  |  |  |
| Intermediate |  | 1.64*** | 2.47*** |  | 0.63*** | 0.71*** |  | 0.89 | 1.03 |
| Low |  | 2.05*** | 4.94*** |  | 0.34*** | 0.43*** |  | 0.80*** | 1.19* |
| Income |  |  |  |  |  |  |  |  |  |
| High | *REF* |  |  |  |  |  |  |  |  |
| Intermediate |  | 1.69*** | 2.14*** |  | 0.89 | 0.76* |  | 0.98 | 1.11 |
| Low |  | 2.42*** | 5.34*** |  | 0.64*** | 0.54*** |  | 1.03 | 1.83*** |
| Occupation |  |  |  |  |  |  |  |  |  |
| High | *REF* |  |  |  |  |  |  |  |  |
| Intermediate |  | 1.28* | 1.25* |  | 0.67** | 0.56*** |  | 0.91 | 0.87 |
| Low |  | 2.33*** | 4.46*** |  | 0.45*** | 0.32*** |  | 0.84 | 1.29* |

Note: *p < .05, **p < .01, ***p < .001.

Table S2. Interactions of age with socioeconomic position indicators and psychosocial work environment.

|  | Prediabetes | Prevalent T2DM | Incident T2DM |
| --- | --- | --- | --- |
| High Education * Age | *REF* |  |  |
| Intermediate Education * Age | 2.03  (0.64, 6.47) | 0.99  (0.97, 1.01) | 1.02  (0.98 1.07) |
| Low Education * Age | 1.95  (0.57, 6.72) | 0.98*  (0.96, 0.99) | 1  (0.95, 1.04) |
| High Income * Age | *REF* |  |  |
| Intermediate Income * Age | 0.47  (0.12, 1.87) | 1  (0.98, 1.02) | 0.96  (0.90, 1.02) |
| Low Income * Age | 1.39  (0.39, 4.95) | 0.99  (0.97, 1.01) | 0.96  (0.91, 1.03) |
| High Occupation * Age | *REF* |  |  |
| Intermediate Occupation * Age | 0.97*  (.94, 1) | 1.02  (.99, 1.05) | 0.97  (0.89, 1.05) |
| Low Occupation * Age | 0.98  (.96, 1.01) | .99  (.96, 1.02) | 1  (0.92, 1.08) |
| High Job Control * Age | *REF* |  |  |
| Intermediate Job Control * Age | 1.00  (0.98, 1.01) | 1.00  (0.98, 1.01) | 0.96  (0.91, 1) |
| Low Job Control * Age | 1.00  (0.98, 1.02) | 0.97**  (0.96, 0.99) | 0.95*  (0.91, 0.99) |
| Low Job Demand * Age | *REF* |  |  |
| Intermediate Job Demand * Age | 0.99  (0.97, 1.02) | 1.00  (0.98, 1.02) | 0.98  (0.93, 1.02) |
| High Job Demand * Age | 1.00  (0.98, 1.02) | 1.00  (0.98, 1.02) | 1.00  (0.95, 1.05) |
| High Job Support * Age | *REF* |  |  |
| Intermediate Job Support * Age | 1.01  (0.99, 1.03) | 1.02  (1.00, 1.03) | 0.96  (0.91, 1) |
| Low Job Support * Age | 1.01  (0.99, 1.03) | 0.99  (0.97, 1) | 0.96  (0.92, 1) |

Note: *p < .05, **p < .01, ***p < .001.

Table S3. Interaction of sex with socioeconomic position indicators and psychosocial work environment.

|  | Prediabetes | Prevalent T2DM | Incident T2DM |
| --- | --- | --- | --- |
| High Education * Sex (M) | *REF* |  |  |
| Intermediate Education * Sex (M) | 0.83  (0.60, 1.14) | 0.77  (0.56, 1.07) | 1.14  (0.51, 2.56) |
| Low Education * Sex (M) | 1.02  (0.75, 1.39) | 0.83  (0.61, 1.12) | 0.98  (0.47. 2.05) |
| High Income * Sex (M) | *REF* |  |  |
| Intermediate Income * Sex (M) | .65*  (.45, .94) | 1.13  (.76, 1.68) | 1.05  (0.35, 3.13) |
| Low Income * Sex (M) | .74  (.53, 1.04) | .87  (.62, 1.22) | 0.71  (0.26, 1.89) |
| High Occupation * Sex (M) | *REF* |  |  |
| Intermediate Occupation * Sex (M) | 1.43  (.89, 2.31) | 1.80*  (1.05, 3.08) | 1.21  (0.33, 4.43) |
| Low Occupation * Sex (M) | 1.26  (.78, 2.02) | 1.65  (.99, 2.75) | 0.93  (0.28, 3.05) |
| High Job Control * Sex (M) | *REF* |  |  |
| Intermediate Job Control * Sex (M) | 0.78  (0.57, 1.07) | 1.01  (0.74, 1.37) | 1.11  (0.52, 2.38) |
| Low Job Control * Sex (M) | 1.03  (0.74, 1.43) | 1.11  (0.82, 1.50) | 0.69  (0.33, 1.47) |
| Low Job Demand * Sex (M) | *REF* |  |  |
| Intermediate Job Demand * Sex (M) | 0.95  (0.68, 1.32) | 1.36  (0.99, 1.86) | 1.13  (0.53, 2.45) |
| High Job Demand * Sex (M) | 0.89  (0.62, 1.26) | 1.02  (0.74, 1.41) | 0.93  (0.42, 2.08) |
| High Job Support * Sex (M) | *REF* |  |  |
| Intermediate Job Support * Sex (M) | 1.01  (0.75, 1.36) | 0.92  (0.69, 1.23) | 1.06  (0.46 2.45) |
| Low Job Support * Sex (M) | 1.01  (0.72, 1.41) | 0.86  (0.63, 1.17) | 0.71  (0.35, 1.45) |

Note: *p < .05, **p < .01, ***p < .001.

Table S4. 2-way and 3-way interactions between psychosocial work stressors.

|  | Prediabetes | Prevalent T2DM | Incident T2DM |
| --- | --- | --- | --- |
| Intermediate Control  * High Demand | 1.24  (0.81, 1.89) | 0.97  (0.66, 1.41) | 1.78  (0.67, 4.75) |
| Intermediate Control  * Intermediate Demand | 1.05  (0.70, 1.57) | 0.92  (0.64, 1.33) | 0.78  (0.30, 1.99) |
| Low Control  * High Demand | 0.94  (0.62, 1.43) | 1.50*  (1.03, 2.19) | 1.59  (0.62, 4.08) |
| Low Control  * Intermediate Demand | 0.85  (0.58, 1.27) | 1.35  (0.93, 1.97) | 0.72  (0.29, 1.80) |
| Intermediate Control  * Low Support | 1.31  (0.85, 2.03) | 1.03  (0.70, 1.52) | 0.78  (0.27, 2.24) |
| Intermediate Control  * Intermediate Support | 1.30  (0.92, 1.84) | 1.17  (0.85, 1.62) | 1.33  (0.47, 3.74) |
| Low Control  * Low Support | 1.12  (0.72, 1.73) | 1.50*  (1.01, 2.23) | 2.33  (0.89, 6.13) |
| Low Control  * Intermediate Support | 0.91  (0.62, 1.35) | 1.21  (0.84, 1.75) | 1.68  (0.55, 5.14) |
| Intermediate Support  * High Demand | 1.33  (0.88, 1.99) | 1.08  (0.74, 1.56) | 0.59  (0.18, 1.90) |
| Intermediate Support  * Intermediate Demand | 1.17  (0.80, 1.70) | 0.99  (0.69, 1.42) | 1.14  (0.39, 3.29) |
| Low Support  * High Demand | 0.98  (0.63, 1.54) | 0.97  (0.65, 1.44) | 1.54  (0.60, 3.99) |
| Low Support  * Intermediate Demand | 1.12  (0.73, 1.72) | 1.09  (0.74, 1.61) | 1.19  (0.45, 3.20) |
| Intermediate Control  * High Demand  * Intermediate Support | 0.63  (0.23, 1.71) | 0.71  (0.29, 1.74) | NA^a^ |
| Low Control  * High Demand  * Intermediate Support | 0.87  (0.30, 2.49) | 0.74  (0.28, 1.96) |  |
| Intermediate Control  * Intermediate Demand  * Intermediate Support | 0.66  (0.26, 1.67) | 1.00  (0.42, 2.36) |  |
| Low Control  * Intermediate Demand  * Intermediate Support | 0.65  (0.24, 1.75) | 0.67  (0.26, 1.75) |  |
| Intermediate Control  * High Demand  * Low Support | 0.63  (0.18, 2.16) | 0.34*  (0.12, 0.94) |  |
| Low Control  * High Demand  * Low Support | 0.64  (0.19, 2.13) | 0.64  (0.22, 1.82) |  |
| Intermediate Control  * Intermediate Demand  * Low Support | 0.52  (0.16, 1.68) | 0.74  (0.26, 2.05) |  |
| Low Control  * Intermediate Demand  * Low Support | 0.56  (0.18, 1.72) | 0.89  (0.31, 2.56) |  |

Note. Reference categories are high job control, low job demand, and high job support.

Note: *p < .05, **p < .01, ***p < .001.

^a^The model does not have enough power for 3-way interaction.
